# Impaired sequence manipulation in non‐demented patients with progressive supranuclear palsy

**DOI:** 10.1002/brb3.3527

**Published:** 2024-05-03

**Authors:** Guanyu Zhang, Jinghong Ma, Piu Chan, Zheng Ye

**Affiliations:** ^1^ China Institute of Sport Science Beijing China; ^2^ Department of Neurology Xuanwu Hospital of Capital Medical University Beijing China; ^3^ Department of Neurobiology, Neurology and Geriatrics, Xuanwu Hospital of Capital Medical University Beijing Institute of Geriatrics Beijing China; ^4^ Institute of Neuroscience, Center for Excellence in Brain Science and Intelligence Technology Chinese Academy of Sciences Shanghai China

**Keywords:** cognitive impairment, levodopa, neuropsychological tests, progressive supranuclear palsy‐Richardson's syndrome, sequential working memory

## Abstract

**Purpose:**

Sequential working memory is the ability to maintain and manipulate sequential information at a second time scale. Patients with progressive supranuclear palsy (PSP) or Parkinson's disease (PD) perform poorly in tests that require the flexible arrangement of thoughts or actions. This study investigated whether sequential working memory is differently impaired in patients with PSP versus PD.

**Method:**

Twenty‐nine patients with PSP Richardson's syndrome (PSP‐RS), 36 patients with PD, and 36 healthy controls (HC) completed 3 well‐established neuropsychological tests, including digit span forward (DST‐F), digit span backward (DST‐B), and adaptive digit ordering tests (DOT‐A). The DST‐F required maintaining digit sequences, and the DST‐B and DOT‐A required maintaining and manipulating digit sequences.

**Finding:**

The PSP‐RS group scored lower than the PD and HC groups in the DST‐B and DOT‐A but not in the DST‐F, indicating that the ability to manipulate sequences was impaired, but the maintenance ability was preserved in PSP‐RS patients. Moreover, in PSP‐RS, the DST‐B score negatively correlated with the severity of motor symptoms. The actual levodopa dose positively correlated with the DST‐B ordering cost (DST‐F score vs. DST‐B score). The PSP patients who took a greater dose of levodopa tended to have higher DST‐B ordering cost. There was no effect of levodopa on DST‐B or DOT‐A in PD.

**Conclusion:**

These results suggested that the ability to manipulate sequence was already reduced in patients with PSP‐RS and was worse than in patients with PD.

## Graphical abstract

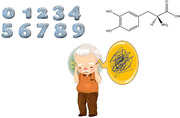

## 1 INTRODUCTION

Sequential working memory refers to the ability to maintain or manipulate sequential information within a short time. Progressive supranuclear palsy (PSP) or Parkinson's disease (PD) patients perform poorly in tests that require the flexible arrangement of thoughts and actions in a specific order. For example, Smith et al. (2021) used the Corsi block task, in which participants were asked to remember the location of a sequence of memoranda within a short period of time, and found that PSP patients had shorter spans compared to healthy adults. PSP patients also have difficulty planning sequential moves (Lange et al., 2003). PD patients at early stages are impaired in understanding the temporal relation of events expressed without chronological order and organizing verbal sequences of digits (Ma et al., 2018; Ye et al., 2012).

As a rare neurodegenerative disease with a prevalence estimate of 1/100,000 from an epidemiological study (Nath, 2001), PSP is characterized by abnormal intracerebral tau deposition with gait disturbance, supranuclear vertical ophthalmoplegia, and cognitive dysfunction. Cognitive impairment even occurs at early stages of PSP (about 50%), which is similar to that reported in idiopathic PD (about 36%) (Brown et al., 2010; Foltynie et al., 2004). Therefore, early detection has important implications for diagnosis and management. Previous studies often included PSP patients at early and advanced stages (Lee et al., 2016; Robbins et al., 1994), so it is unclear whether the deficits in sequential working memory occur at early stages of PSP. PSP Richardson's syndrome (PSP‐RS) is the most prevalent clinical phenotype of PSP with the highest rate of dementia and the most extensive dysfunction in cortical and subcortical networks (Picillo et al., 2019).

In clinical practice, three neuropsychological tests with verbal responses could be readily used to assess sequential working memory. Digit span tests (DST) from the Wechsler Adult Intelligence Scale include the digit span forward test (DST‐F) and digit span backward test (DST‐B) (Jasinski et al., 2011; Wechsler, 1945). As a quick, simple, and inexpensive test, the DST can be applied to support the differential diagnosis of PSP (Vaccaro et al., 2021). Moreover, DST has been shown to be sensitive to detect cognitive progression in PSP‐RS (Fiorenzato et al., 2019). The adaptive digit ordering test (DOT‐A) developed in analogy to the DST is a promising diagnostic instrument due to its high sensitivity for patients with PD and patients with frontal lobe damage (Werheid et al., 2002).

In this study, we assessed the sequential working memory of PSP‐RS patients at early stages. Moreover, we explored the relationship between the ability to maintain and manipulate information, and the effect of levodopa on sequential working memory in PSP. All participants completed three sequential working memory tests, including DST‐F, DST‐B, and DOT‐A. We used the DST‐F to evaluate the maintenance of sequential digits, and the DST‐B and DOT‐A to evaluate the maintenance and manipulation of sequential digits for different ordering rules (reversing and ascending). First, we examined group differences in test scores and ordering costs (score difference between the DST‐F and DST‐B/DOT‐A). Second, in PSP‐RS, we explored whether the test scores or ordering costs correlated with the severity of motor or non‐motor symptoms. Third, in PSP‐RS, we investigated the effect of levodopa on test scores and ordering costs using correlation tests.

## 2 MATERIALS AND METHODS

This study was approved by the ethics committee of the Xuanwu Hospital according to the Declaration of Helsinki. Each participant signed a written informed consent before participating in this study.

The methods of this study are similar to our recent study (Ma et al., 2023). Our recent study aimed to assess the PSP‐RS patients’ semantic fluency, but this study aimed to assess the PSP‐RS patients’ sequential working memory.

### 2.1 Patients and clinical assessments

We included 29 patients with probable PSP‐RS (Movement Disorder Society [MDS] Clinical Diagnostic Criteria for PSP (Höglinger et al., 2017)) at the Xuanwu Hospital between 2022 and 2023. Inclusion criteria were (1) age 40–80 years; (2) education ≥6 years; (3) Hoehn and Yahr Stages 1–3; and (4) Mandarin Chinese speaking. Exclusion criteria were (1) alcohol or drug abuse; (2) a history of epilepsy, stroke, or brain injury; (3) possible current dementia (Montreal Cognitive Assessment, MoCA < 21/30) or intake of anti‐dementia drugs; (4) possible current depression (Beck Depression Inventory‐II >7) or intake of anti‐depressants.

All patients were parkinsonian and had no response to levodopa. They were assessed on their regular anti‐parkinsonian drugs, including levodopa (*N* = 22), selegiline (*N* = 5), amantadine (*N* = 3), piribedil (*N* = 3), pramipexole (*N* = 3), and rasagiline (*N* = 3). The levodopa equivalent daily dose was calculated using the equation of Tomlinson et al. (2010). The severity of non‐motor and motor symptoms was evaluated with the Non‐Motor Symptoms Scale (NMSS) and the MDS‐sponsored revision of the Unified PD rating scale (MDS‐UPDRS) Part III subscale, respectively. Given that PSP is often associated with a deficit of speech production, we compared the subscore of speech from MDS‐UPDRS Part III and found no difference between PSP‐RS and PD groups (*t *< 1). Table 1 shows demographic and clinical features, and neuropsychological measures. There was no difference between PSP‐RS and PD groups (*p *= .154) in MoCA, although there were significant differences among the three groups.

**TABLE 1:** Demographic and clinical features and neuropsychological measures of patients and healthy controls (means, standard deviations, and group differences).

Features/Measures	PSP‐RS (*N* = 29)	PD (*N* = 36)	Healthy controls (*N* = 36)	Group differences (*p* values)
Male:Female	14:15	19:17	19:17	.920
Age (years)	62.1 (6.2)	61.6 (8.8)	59.0 (8.7)	.235
Education (years)	10.7 (3.0)	11.6 (3.2)	11.9 (2.9)	.269
Disease duration (years)	1.3 (2.2)	2.0 (2.0)	–	.230
*Motor symptoms*				
MDS‐UPDRS III: Motor examination	28.8 (11.8)	27.1 (14.3)	–	.618
Hoehn and Yahr scale	2.4 (0.6)	2.1 (0.7)	–	.065
Duration of motor symptoms (years)	2.7 (2.1)	3.5 (2.6)	–	.217
*Levodopa equivalent daily dose*				
Total (mg/day)	320.7 (252.7)	327.3 (258.6)	–	.917
Levodopa (mg/day)	257.8 (193.1)	248.5 (212.9)	–	.857
*Non‐motor functions*				
Non‐Motor Symptoms Scale	38.5 (24.2)	26.0 (22.5)	–	.125
Beck Depression Inventory‐II	3.7 (2.0)	3.1 (2.1)	2.5 (2.0)	.072
Epworth Sleep Scale	5.1 (5.2)	2.7 (2.5)	3.7 (2.8)	.141
Montreal Cognitive Assessment	23.2 (2.1) ^a^	24.0 (2.2) ^a^	27.3 (1.3)	<.001*

*Note*: PSP‐RS, progressive supranuclear palsy‐Richardson's syndrome; PD, Parkinson's disease; MDS‐UPDRS, the Movement Disorder Society–sponsored revision of the unified Parkinson's disease rating scale; group differences, *p* values of one‐way ANOVAs or Kruskal–Wallis one‐way ANOVAs as appropriate; asterisks (*), a significant difference (two‐tailed, *p *< .004 Bonferroni correction for 13 tests); post‐hoc two‐sample *t*‐tests, *p *< .004.

^a^ Compared with healthy controls.

### 2.2 Two control groups

We included two control groups: 36 age‐ and education‐matched patients with idiopathic PD (MDS Clinical Diagnostic Criteria for PD (Postuma et al., 2015)) from Xuanwu Hospital and 36 age‐ and education‐matched healthy controls (HC) from local communities.

The inclusion and exclusion criteria of PD group were same as for PSP‐RS. They were assessed on their regular anti‐parkinsonian drugs, including levodopa (*N* = 27), pramipexole (*N* = 10), piribedil (*N* = 8), amantadine (*N* = 4), selegiline (*N* = 4), and entacapone (*N* = 3). They completed the same clinical and neuropsychological measures as patients with PSP‐RS.

For the HC group, exclusion criteria were (1) alcohol or drug abuse; (2) a history of significant neurological or psychiatric disorders; (3) possible dementia or mild cognitive impairment (MoCA < 26/30); (4) possible current depression. They completed the same measures for cognition, mood, and sleep as patients.

### 2.3 Sequential working memory tests

All participants completed three sequential working memory tests, including DST‐F, DST‐B, and DOT‐A. Participants heard a series of random digits at a speed of one digit per second in each trial and immediately recalled the digits in original order in DST‐F, in reversed order in DST‐B, and in ascending order in DOT‐A. Each test started at a three‐digit trial and was terminated when participants incorrectly recalled in both trials of a certain length. For the DST‐F and DST‐B, we scored the length of last successfully recalled trial (span). For the DOT‐A, we scored the number of successfully recalled trials.

### 2.4 Statistical analysis

Data were analyzed with IBM SPSS Statistics 20. First, we detected group differences in test scores and ordering costs by one‐way analyses of variance (ANOVAs) (two‐tailed, *p *< .010 Bonferroni correction for five tests). The ordering cost was defined as score difference between the DST‐F and DST‐B/DOT‐A. The ANOVA had a factor group (PSP‐RS, PD, and HC) and covariates age and education. Significant differences were followed by two‐sample *t*‐tests. In addition, given that the DST‐F and DST‐B were scored using a span measure, we use a 2 × 3 ANOVA with a within‐subject factor test (DST‐F, DST‐B) and a between‐subject factor group (PSP‐RS, PD, and HC) to detect group differences in test scores as a validity check.

Second, in the PSP‐RS group, we detected whether the severity of non‐motor or motor symptoms (NMSS score or MDS‐UPDRS Part III score) correlated with the test scores or ordering costs that showed group differences by linear stepwise regression models (two‐tailed, *p *< .025 Bonferroni correction for two models).

Given the correlation between MDS‐UPDRS Part III score and DST‐B score, third, we examined the effect of levodopa by correlating the actual levodopa dose with DST‐B score in PSP‐RS (two‐tailed, *p *< .05). As a validity check, we then correlated the actual levodopa dose with DST‐B ordering cost (one‐tailed, *p *< .05). In addition, we examined the effect of levodopa on the DST‐B and DOT‐A scores and ordering costs PD patients (two‐tailed, *p *< .05).

## 3 RESULTS

### 3.1 Group differences in test scores and ordering costs

**FIGURE 1:**
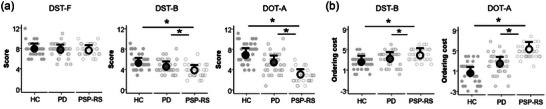
(a) Individual data, group means, and standard errors of digit span forward test (DST‐F), digit span backward test (DST‐B), and adaptive digit ordering test (DOT‐A) scores in patients with progressive supranuclear palsy‐Richardson's syndrome (PSP‐RS), patients with Parkinson's disease (PD), and healthy controls (HC). *p < .05. (b) Individual data, group means, and standard errors of DST‐B and DOT‐A ordering costs in each group. *p < .05.

Figure 1a shows test scores in each group. One‐way ANOVAs revealed significant group differences in the DST‐B (*F*(2, 96) = 9.14, *p <* .001, *η_p_* ^2^ = .16) and DOT‐A (*F*(2, 96) = 42.84, *p <* .001, *η_p_* ^2 ^= .47), but not in the DST‐F (*F* < 1). The 2 × 3 ANOVA revealed a significant interaction between test and group (*F*(2, 98) = 11.37, *p* *<* .001, *η_p_* ^2^ = .19), in addition to the main effects of test (*F*(1, 98) = 575.32, *p* *<* .001, *η_p_* ^2^ = .85) and group (*F*(2, 98) = 4.81, *p = *.010, *η_p_* ^2^ = .09). The PSP‐RS group scored lower than the PD and HC groups in the DST‐B (PD: *t*(63) = −2.53, *p = *.014; HC: *t*(63) = −4.87, *p <* .001) and DOT‐A (PD: *t*(63) = −6.21, *p <* .001; HC: *t*(63) = −10.80, *p <* .001).

Figure 1b shows ordering costs in each group. Significant group differences were found in the DST‐B (*F*(2, 96) = 11.66, *p <* .001, *η_p_* ^2^ = .20) and DOT‐A ordering costs (*F*(2, 96) = 43.46, *p <* .001, *η_p_* ^2^ = .48). The PSP‐RS group had higher DST‐B (PD: *t*(63) = 2.43, *p = *.018; HC: *t*(63) = 5.17, *p* *<* .001) and DOT‐A ordering costs than the PD and HC groups (PD: *t*(63) = 5.98, *p* *<* .001; HC: *t*(63) = 10.03, *p* *<* .001). It means that PSP‐RS patients’ ability to manipulate digits was impaired regardless of the ordering rule, but their maintenance ability was preserved.

### 3.2 Correlations between the severity of motor and non‐motor symptoms and test scores in PSP‐RS

**FIGURE 2:**
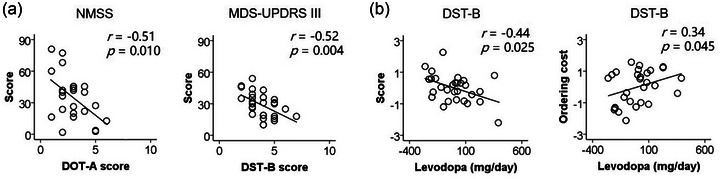
In progressive supranuclear palsy‐Richardson's syndrome (PSP‐RS), (a) the digit ordering test‐A (DOT‐A) score negatively correlated with the severity of non‐motor symptoms scale (NMSS score) and the digit span backward test (DST‐B) score negatively correlated with the severity of motor symptoms Movement Disorder Society–unified Parkinson's disease rating scale (MDS‐UPDRS part III score). (b) The actual levodopa dose negatively correlated with the DST‐B score and positively correlated with DST‐B ordering cost when the levodopa equivalent dose for other drugs, age, and education were controlled. The unstandardized residuals for data were used, and values were demeaned.

Figure 2a shows correlations between the severity of motor and non‐motor symptoms and test scores in PSP‐RS. The stepwise regression model for the MDS‐UPDRS Part III score (*F*(1, 26) = 9.81, *p *= .004, *R* ^2^ = .27) included the DST‐B score (beta = −5.12, *t *= −3.13, *p *= .004) but removed the DOT‐A score (|*t| *< 1), DST‐B ordering cost (|*t| *< 1), and DOT‐A ordering cost (|*t| *< 1). PSP‐RS patients who exhibited more severe motor symptoms tended to perform worse in the DST‐B.

The stepwise regression model for the NMSS score (*F*(1, 22) = 7.92, *p *= .010, *R* ^2 ^= .27) included the DOT‐A score (beta = −9.11, *t *= −2.81, *p *= .010) but removed the DST‐B score (|*t| *< 1), DST‐B ordering cost (beta = .29, *t *= 1.64, *p *= .115), and DOT‐A ordering cost (beta = .36, *t *= 1.56, *p *= .134). PSP‐RS patients who exhibited more severe non‐motor symptoms tended to perform worse in the DOT‐A.

### 3.3 Effect of levodopa on DST‐B ordering cost in PSP‐RS

Figure 2b shows the effect of levodopa on DST‐B score and DST‐B ordering cost in PSP‐RS. After observing the correlation between the actual levodopa dose and the DST‐B score (*r *= −.44, *p *= .025), we found that the actual levodopa dose positively correlated with DST‐B ordering cost (*r *= .34, *p *= .045) when the levodopa equivalent dose for other drugs, age, and education were controlled. The patients who took a greater dose of levodopa tended to have higher DST‐B ordering cost. Levodopa may impair the ability to manipulate sequences in PSP‐RS.

We did not find the effect of levodopa on DST‐B or DOT‐A in PD patients (*p*s > .099).

## 4 DISCUSSION

In this study, we evaluated sequential working memory in non‐demented patients with PSP‐RS. We examined the ability to maintain and manipulate sequences and found that PSP‐RS patients scored lower in DST‐B and DOT‐A and had higher DST‐B and DOT‐A ordering costs than the PD patients and healthy adults. But there was no difference among three groups in DST‐F score. In other words, the ability to manipulate sequences was impaired, but the maintenance of sequences was preserved in PSP‐RS. Moreover, in PSP‐RS, the DOT‐A score and DST‐B score negatively correlated with the severity of motor and non‐motor symptoms, respectively. The PSP‐RS patients who performed worse in DST‐B exhibited more severe motor symptoms, and those who performed worse in DOT‐A exhibited more severe non‐motor symptoms. Importantly, in PSP‐RS, the actual levodopa dose positively correlated with DST‐B ordering cost, indicating that levodopa may impair the manipulation of sequences in PSP‐RS patients.

PSP's sequential deficits were reported in previous studies but were usually attributed to executive dysfunction or attention/working memory damages (Goel & Vyas, 2019; Smith et al., 2021). Nevertheless, the characteristic was not specified. This study proposed that PSP‐RS patients at the early stages showed a selective deficit in the manipulation of sequences. As the main cognitive component of working memory, manipulation and updating should be noticeable in PSP‐RS.

Most existing computational models for sequential working memory emphasize the encoding and retrieval of the original sequence (Hurlstone et al., 2014; Page & Norris, 1998). For example, the competitive queuing model contains a parallel planning layer, which represents the to‐be‐performed items with different activation gradients, and a competitive choice layer, which chooses the item with the strongest activation from the parallel planning layer (Kornysheva et al., 2019). The recall of sequential information is realized by iteration. At each iteration, the item with the strongest activation is first recalled and then inhibited so that the second strongest item is chosen at the next iteration. Electrophysiological studies supported that the prefrontal cortex provides a basis for the competitive queuing model (Averbeck et al., 2002; Berdyyeva & Olson, 2010).

Hazy et al. (2006) argued that a dynamic gating mechanism is needed to switch between updating and maintenance. When the gate is open for incoming information, currently active working memory content can get updated; when it is closed to inhibit distractor stimuli, working memory content are robustly maintained. The basal ganglia play an important role in this dynamic gating mechanism. The profound manipulation impairment in PSP‐RS patients may be due to more severe deficits in frontal–basal ganglionic circuits compared to PD patients (Behari et al., 2008). A functional magnetic resonance imaging study used force production paradigm and showed that the frontal cortex and basal ganglia are underactive in PSP patients than in PD patients (Burciu et al., 2015). Future studies should explore the relationship between sequence manipulation and frontal‐basal ganglionic circuits in PSP patients. Furthermore, it is meaningful to examine the difference between PSP and PD patients in manipulation‐induced activity and functional connectivity of frontal cortex and basal ganglia by neuroimaging.

Previous studies have confirmed the correlation between motor symptoms and cognition in PSP. Soliveri et al. (2005) found that the cognitive ability (the Mini Mental State Examination) was correlated with limb apraxia (Lange et al., 2003). Bak (2006) found that the degree of visuospatial dysfunction correlated with disease duration and the severity of eye movement abnormalities. Our findings were consistent with previous studies. The severity of motor and non‐motor symptoms correlated with DST‐B score and DOT‐A score, respectively.

Neurochemical mechanisms of sequential working memory are still unclear. Dual‐state theory pointed out that dopamine mediates the visuospatial working memory via D1‐ and D2‐class receptors (Durstewitz & Seamans, 2008). To be specific, D1‐dominated state with a high energy barrier is beneficial for the maintenance of information and D2‐dominated state with a low energy barrier is beneficial for the manipulation of information. Dual‐state theory is supported by many studies about sequential working memory. Human psychopharmacological studies reported that levodopa impaired the accuracy of DOT in healthy adults (Grogan et al., 2018). The PD patients who took a greater dose of dopamine D2/3 receptor agonists tended to perform better in DOT‐A (Ma et al., 2019). Even though there are conflicting findings (Brusa et al., 2003; Cooper et al., 1992), we find that levodopa may be harmful to manipulate sequences, perhaps due to an overdosing of intact brain areas (Cools & D'esposito, 2011). In addition, maintenance and manipulation may need different optimal levels of dopamine.

This study has limitations. First, previous studies showed that neuropsychological tests could distinguish among different clinical phenotypes of PSP (Picillo et al., 2019; Vaccaro et al., 2021). We only include patients with PSP‐RS for homogeneity; thus, this study could not compare the sequential working memory performance of PSP patients with different phenotypes. Second, according to the dual‐state theory and previous studies, dopamine D2/3 receptor agonists may have beneficial effects on sequential manipulation (Durstewitz & Seamans, 2008; Ma et al., 2019). This study cannot examine this issue due to small sample size (only six PSP‐RS patients took dopamine D2/3 receptor agonists). Future pharmacological studies can explore the influence of dopamine D2/3 receptor agonists on PSP patients’ sequential working memory.

## 5 CONCLUSION

In this study, we used three well‐established neuropsychological tests to assess the sequential working memory in patients with PSP‐RS. PSP‐RS patients’ ability to manipulate sequences was impaired, but their ability to maintain sequences was preserved. Moreover, PSP‐RS patients who performed worse in the DST‐B exhibited more severe motor symptoms, and those who performed worse in the DOT‐A exhibited more severe non‐motor symptoms. Importantly, levodopa may impair the manipulation of sequences in PSP‐RS.

## AUTHOR CONTRIBUTIONS

Guanyu Zhang was responsible for data curation, formal analysis, and writing the original draft. Jinghong Ma was responsible for investigation, reviewing, and editing the manuscript. Piu Chan was responsible for funding acquisition, reviewing, and editing the manuscript. Zheng Ye was responsible for conceptualization, funding acquisition, and reviewing and editing the manuscript. All authors approved the submitted version.

## CONFLICT OF INTEREST STATEMENT

The authors declared no conflicts of interest.

### PEER REVIEW

The peer review history for this article is available at https://publons.com/publon/10.1002/brb3.3527.

## Data Availability

The data used to support the findings of this study are included in the manuscript. Further information is available from the corresponding authors upon request.
